# Predictive value of the stress hyperglycemia ratio in patients with acute ST-segment elevation myocardial infarction: insights from a multi-center observational study

**DOI:** 10.1186/s12933-022-01479-8

**Published:** 2022-03-29

**Authors:** Wei Xu, Yan-min Yang, Jun Zhu, Shuang Wu, Juan Wang, Han Zhang, Xing-hui Shao

**Affiliations:** 1 Emergency Center, National Center for Cardiovascular Disease, Fuwai Hospital, Chinese Academy of Medical Sciences and Peking Union Medical College, No. 167 Beilishi Road, Xicheng District, Beijing, People’s Republic of China; 2 National Clinical Research Center of Cardiovascular Diseases, National Center for Cardiovascular Disease, Fuwai Hospital, Chinese Academy of Medical Sciences and Peking Union Medical College, Beijing, China

**Keywords:** Stress hyperglycemia ratio, ST-segment elevation myocardial infarction, Diabetes mellitus

## Abstract

**Background:**

Stress hyperglycemia is a strong predictor of adverse outcomes in patients with acute myocardial infarction (AMI). Recently, the stress hyperglycemia ratio (SHR) has been designed as an index to identify acute hyperglycemia with true risk; however, data regarding the impact of SHR on the prognosis of ST-segment elevation myocardial infarction (STEMI) remains limited. This study aimed to evaluate the predictive value of the SHR in patients with acute STEMI and to assess whether it can improve the predictive efficiency of the Thrombolysis in Myocardial Infarction (TIMI) risk score.

**Methods:**

This study included 7476 consecutive patients diagnosed with acute STEMI across 274 emergency centers. After excluding 2052 patients due to incomplete data, 5417 patients were included in the final analysis. Patients were divided into three groups according to SHR tertiles (SHR1, SHR2, and SHR3) and were further categorized based on diabetes status. All patients were followed up for major cardiovascular adverse events (MACEs) and all-cause mortality.

**Results:**

After 30 days of follow-up, 1547 MACEs (28.6%) and 789 all-cause deaths (14.6%) occurred. The incidence of MACEs was highest among patients in the SHR3 group with diabetes mellitus (DM) (42.6%). Kaplan–Meier curves demonstrated that patients with SHR3 and DM also had the highest risk for MACEs when compared with other groups (p < 0.001). Moreover, C-statistics improved significantly when SHR3 was added into the original model: the ΔC-statistics (95% confidence interval) were 0.008 (0.000–0.013) in the total population, 0.010 (0.003–0.017) in the DM group, and 0.007 (0.002–0.013) in the non-DM group (all p < 0.05). In the receiver operating characteristic analysis, the area under the curve (AUC) for the original TIMI risk score for all-cause death was 0.760. When an SHR3 value of 1 point was used to replace the history of DM, hypertension, or angina in the original TIMI risk score, the Delong test revealed significant improvements in the AUC value (∆AUC of 0.009, p < 0.05), especially in the DM group (∆AUC of 0.010, p < 0.05).

**Conclusion:**

The current results suggest that SHR is independently related to the risks of MACEs and mortality in patients with STEMI. Furthermore, SHR may aid in improving the predictive efficiency of the TIMI risk score in patients with STEMI, especially those with DM.

**Supplementary Information:**

The online version contains supplementary material available at 10.1186/s12933-022-01479-8.

## Background

Ischemic heart disease represents the most common cause of death worldwide [1]. While increases in the use of reperfusion therapy, percutaneous coronary intervention (PCI), and secondary prevention therapy have decreased the mortality of ST-segment elevation myocardial infarction (STEMI), the incidence of in-hospital mortality remains high at 4–12% [1, 2]. Stress hyperglycemia—defined as a transient increase in blood glucose related to the stress of illness, is a strong predictive factor for adverse outcomes in patients with acute myocardial infarction (AMI) [3, 4], including those with non-obstructive coronary arteries (MINOCA) [5]. To explain this phenomenon, researchers have speculated that stress hyperglycemia caused by sympathetic system activation, leads to oxidative stress and endothelial dysfunctions [6]. Thus, acute glucose evaluation is considered more effective than chronic hyperglycemia status for predicting the prognosis of STEMI.

To distinguish whether the evaluated admission blood glucose (ABG) levels represented acute or chronic glucose elevation, Robert et al. proposed the novel stress-hyperglycemia ratio (SHR), which is calculated by taking the ratio of ABG to estimated blood glucose. The authors reported that SHR is an effective predictor of adverse events in patient with critical illness [7]. Further studies have demonstrated that the SHR exhibits better predictive value than ABG in cases of AMI [8, 9]. However, data regarding the effect of SHR on the prognosis of STEMI remain limited. Proposed in 2000, the Thrombolysis in Myocardial Infarction (TIMI) risk score for STEMI includes diabetes mellitus (DM) as a risk factor without considering the influence of acute hyperglycemia. Thus, it is unclear whether the SHR exhibits predictive value independent of the traditional TIMI risk score. This study aimed to evaluate the predictive value of the SHR for major cardiovascular adverse events (MACEs) and all-cause mortality in patients with STEMI and to assess whether it can improve the predictive efficiency of the TIMI risk score.

## Methods

### Study design and population

**Fig. 1:**
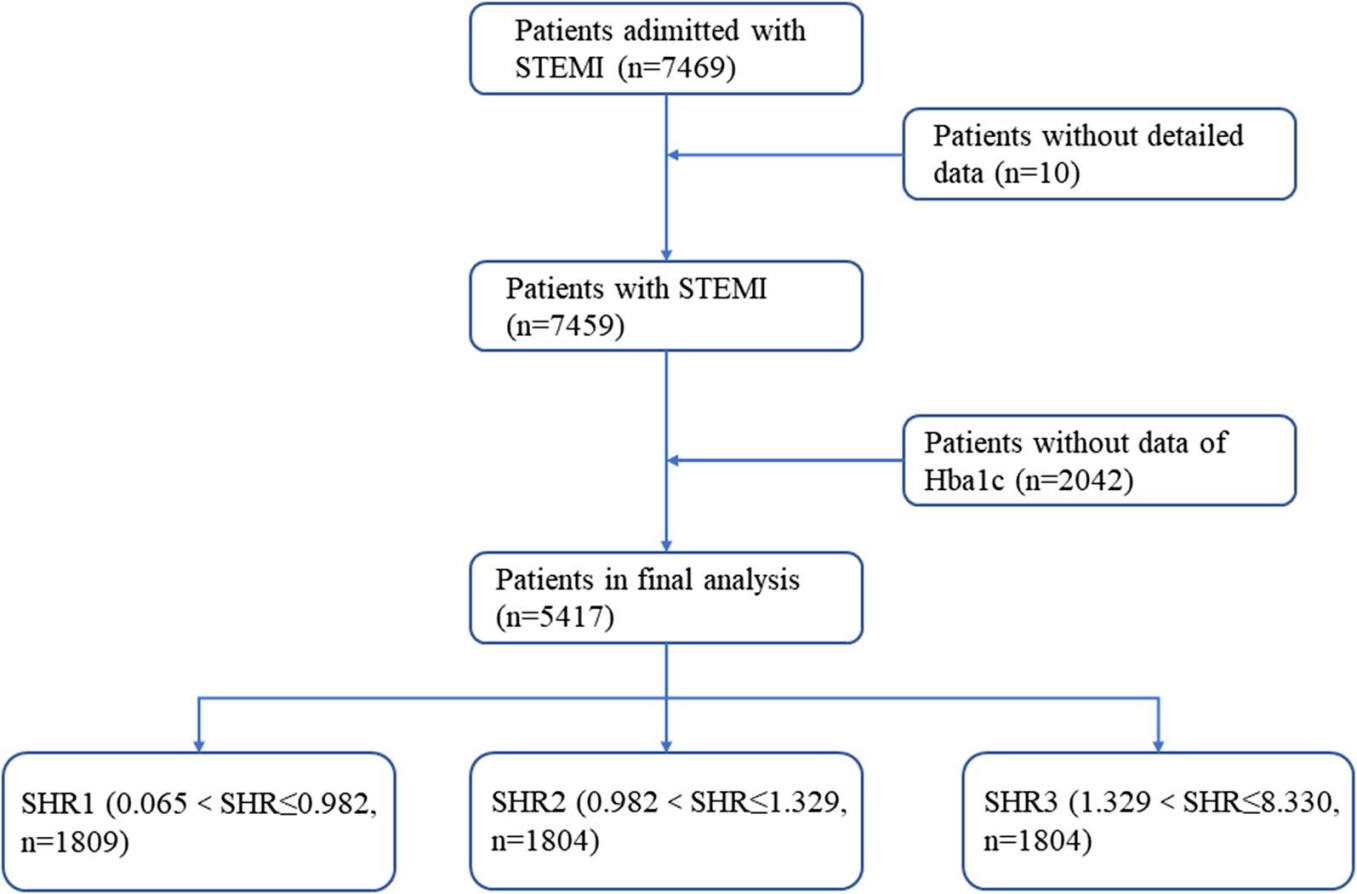
Study flowchart. *STEMI* ST-segment elevation myocardial infarction, *SHR* stress hyperglycemia ratio

This multi-center observational study was conducted across 274 centers in China. A total of 7476 consecutive patients with acute STEMI admitted within 12 h following an attack between June 2001 to July 2004 were included. The diagnostic criteria for acute STEMI were as follows: (1) typical chest pain or ischemia symptoms; (2) dynamic changes in the new electrocardiogram: ST-segment elevation of more than two adjacent leads (V1, V2, and V3 leads of ≥ 0.2 mV, other leads of ≥ 0.1 mV) or new left bundle branch block (LBBB); (3) increased levels of biochemical markers of myocardial injury (troponin and creatine kinase MB) [10]. The exclusion criteria were the presence of anticoagulant contraindications, hemorrhagic stroke within the past 12 months, pregnancy, malignancy, and inability to complete expected follow-up. After admission, patients received reperfusion therapy including primary PCI or thrombolytic therapy based on clinical guidelines applicable during the study period and local healthcare levels. Primary PCI was performed via radial or femoral artery access in accordance with the standard techniques by cardiologists at experienced centers. Patients were administered aspirin (300 mg) or clopidogrel (300 mg) before primary PCI. During the PCI procedure, patients received systemic anticoagulation with unfractionated heparin or low molecular weight heparin (LMWH). As the flowchart in Fig. 1 shows, 10 patients without detailed data and 2042 patients lacking the laboratory results for HbA1c were excluded. Thus, 5417 patients were included in the final analysis. Patients were divided into three groups according to SHR tertiles. This study complied with the principles outlined in the Declaration of Helsinki and was approved by the Ethics Committee of each center. All the patients have provided written informed consent.

### Definitions

DM was defined as an HbA1c levels of ≥ 6.5%, while non-DM was defined as an HbA1c levels of < 6.5%. ABG was determined based on blood glucose levels of patients in 6 ± 2 h upon admission. The SHR was calculated as ABG divided by the estimated average glucose level [7]. Estimated average glucose was calculated by the following formula: estimated average glucose (mmol/L) = 1.59 × HbA1c (%) − 2.59. Accordingly, the SHR was calculated as follows: SHR = ABG/ [1.59 × HbA1c(%) − 2.59].

### Laboratory measurements

To assess ABG, blood samples were obtained from the cubital vein of each patient at a mean of 6 ± 2 h after admission and analyzed using the glucose oxidase (GOD) method. All centers were required to use the same method to assess ABG. Blood samples for testing HbA1c levels were obtained from the cubital vein within 24 ± 4 h of admission and transferred to the central laboratory (Fuwai Hospital) located in Beijing for testing using the high-pressure liquid chromatography (HPLC) method.

### Endpoints and follow up

The endpoints of this study were major adverse cardiovascular events (MACEs) and all-cause mortality. MACEs were composite endpoints and included all-cause death, cardiogenic shock, cardiac arrest, recurrent myocardium infarction, malignant arrhythmia, heart failure, and stroke. All patients were followed up for 30 days through telephone interviews, outpatient follow-up, or a review of medical records. The endpoint events were assessed by well-trained physicians who were blinded to the study objective.

### Statistical analysis

Continuous variables were expressed as means ± standard deviation (SD) or medians (25–75%) based on the normality of the distribution, which was assessed using the Kolmogorov–Smirnov test. Differences between groups were analyzed using the independent t-test or Kruskal–Wallis test based on the distribution of continuous variables. Categorical variables were presented as numbers (percentages) and were compared using Pearson’s chi-square test or Fisher’s exact test. Univariable and multivariable Cox proportional hazards regression models were used to calculate hazard ratios (HRs). Other candidate risk factors included systolic blood pressure (SBP), heart rate (HR), Killip classification, DM, hypertension, angina, weight, anterior ST-segment elevation or LBBB, and time to treatment > 4 h (factors derived from the TIMI risk score). The predictors that reached a significance level of p < 0.10 were used for adjustment in the multivariate analysis. Kaplan–Meier survival curves were generated to evaluate the incidence of MACEs within different subgroups and compared by Log-rank test. C-statistics and ΔC-statistics were calculated to assess the predictive value of SHR3. Moreover, the area under the curve (AUC) and ΔAUC were calculated to evaluate the efficiency of the adjusted TIMI score, in which SHR3 was used to replace history of DM, hypertensive or angina (value of 1 point). The receiver operating characteristic (ROC) curves were compared by the Delong test. The Hosmer–Lemeshow goodness-of-fit test was used to assess the calibration of the models. Statistical analysis was performed using with SPSS version 26.0 (SPSS Inc., Chicago, IL, USA) and the R version 4.0.4. p < 0.05 was considered statistically significant, and all the analyses were two-tailed.

## Results

### Baseline characteristics

A total of 5417 patients with acute STEMI were included in the final analysis. The median age of the study population was 65 (54–72) years, and 1641 (30.9%) patients were women.

Patients were divided into three groups according to the SHR tertiles (Fig. 1). Table 1 shows the baseline characteristics of the study population according to the SHR tertiles. Older age, female sex, hypertension, DM, and history of stroke were most frequent in the highest tertile (SHR3 group). Lower blood pressure, higher heart rates, higher Killip classes, and higher TIMI risk score were also more likely in the SHR3 group than in the other groups. ABG values were higher, while HbA1c levels were lower, in the SHR3 group than in the other two groups.

**Table 1:** Baseline characteristics according to different SHR tertiles

Variables	Total	SHR tertiles			p-value
		SHR1 (≤ 0.982)	SHR2 (0.982–1.329)	SHR3 (≥ 1.329)	
n	5417	1809	1804	1804	
Clinical characteristics					
Age (years)	65 (54–72)	64 (53–71)	65 (55–72)	65 (56–72)	< 0.001
Female,n (%)	1641 [30.29%]	502 [27.75%]	489 [27.11%]	650 [36.03%]	< 0.001
Weight (Kg)	65 (60–70)	65 (60–75)	67 (60–75)	65 (60–75)	0.072
SBP (mmHg)	125 (110–140)	125 (110–140)	128 (110–142)	120 (105–140)	< 0.001
DBP (mmHg)	80 (70–90)	80 (70–90)	80 (70–90)	80 (69–90)	0.006
HR (beats/min)	76 (64–88)	76 (64–86)	75 (64–86)	77 (64–90)	0.001
Killip					< 0.001
1	4415 [81.50%]	1522 [84.13%]	1519 [84.20%]	1374 [76.16%]	
2	648 [11.96%]	197 [10.89%]	200 [11.09%]	251 [13.91%]	
3	199 [3.67%]	56[3.10%]	48 [2.66%]	95 [5.27%]	
4	155 [2.86%]	34 [1.88%]	37 [2.05%]	84 [4.66%]	
Angina	442 [8.16%]	140 [7.74%]	152 [8.43%]	150 [8.31%]	0.721
Anterior STE or LBBB	2862 [52.83%]	989 [54.67%]	941 [52.16%]	932 [51.66%]	0.152
Hypertension	2177 [40.19%]	676 [37.37%]	727 [40.30%]	775 [42.96%]	0.003
Diabetes mellitus	1336 [24.66%]	627 [34.66%]	296 [16.41%]	413 [22.89%]	< 0.001
Prior stroke	520 [9.60%]	145 [8.02%]	172 [9.53%]	203 [11.25%]	0.004
TIMI class					< 0.001
1	2415 [44.58%]	849 [46.93%]	863 [47.84%]	703 [38.96%]	
2	2113 [39.01%]	695 [38.42%]	691 [38.30%]	727 [40.30%]	
3	889 [16.41%]	265 [14.65%]	250 [13.86%]	374 [20.73%]	
Laboratory tests					
Glucose(mmol/L)	7.4 (6.0–9.8)	5.7 (5.0–6.4)	7.2 (6.5–8.2)	11 (9.0–14.9)	< 0.001
HbA1c(%)	5.7 (5.3–6.4)	6.0 (5.6–6.8)	5.6 (5.3–6.1)	5.6 (5.2–6.3)	< 0.001
Hemoglobin(g/L)	135 (124–148)	136 (124–147)	136 (124–148)	135 (122–147)	0.406
Reperfusion therapy					
Primary PCI	691 [12.76%]	166 [9.18%]	251 [13.91%]	274 [15.19%]	< 0.001
Thrombolytic therapy	2747 [50.71%]	880 [48.65%]	913 [50.61%]	954 [52.88%]	0.039
Culprit lesion					0.041
LAD Artery	382 [7.05%]	97 [5.36%]	136 [7.54%]	149 [8.26%]	
RCA Artery	239 [4.41%]	50 [2.76%]	81 [4.49%]	108 [5.99%]	
LCX Artery	70 [1.30%]	18 [1.00%]	34 [1.88%]	18 [1.00%]	
Medications					
Antiplatelet therapy	5225 [96.46%]	1730 [95.63%]	1757 [97.39%]	1738 [96.34%]	0.016
Statins	3927 [72.49%]	1338 [73.96%]	1328 [73.61%]	1261 [69.90%]	0.010
β-blockers	2954 [54.53%]	1102 [60.92%]	1167 [64.69%]	1087 [60.25%]	0.013
ACEI/ARB	3874 [71.52%]	1312 [72.53%]	1306 [72.39%]	1256 [69.62%]	0.092
Diuretics	758 [13.99%]	213 [11.77%]	216 [11.97%]	329 [18.24%]	< 0.001

*ABG* admission blood glucose, *ACEI* angiotensin-Converting Enzyme Inhibitors, *ARB* Angiotensin Receptor Blocker, *DBP* diastolic blood pressure, *HR* heart rate, *LAD* left anterior descending, *LCX* left circumflex, *RCA* right coronary artery, *SBP* systolic blood pressure, *SHR* stress hyperglycemia ratio, *STE* ST-segment elevation, *LBBB*, left bundle-branch block

### Clinical outcomes according to SHR tertiles and diabetes status

Over the 30 days of follow-up, a total of 1547 MACEs (28.6%) occurred, while 789 all-cause deaths (14.6%) occurred. As shown in Table 2, when compared with the SHR1-2 group, the SHR3 group exhibited an increased incidence of MACEs and all-cause mortality in the analyses covering the total study population, those with DM, and those with non-DM (p < 0.001).

**Table 2:** Associations between SHR3 and clinical outcomes

				Univariable analysis			Multivariable analysis*	
	SHR1-2	SHR3	p-value	HR (95% CI)	p-value		HR (95% CI)	p-value
Total population								
MACEs	867 [24.00%]	680 [37.69%]	< 0.001	1.694 (1.532–1.873)	< 0.001		1.416 (1.265–1.584)	< 0.001
All-cause death	506 [14.00%]	283 [15.69%]	< 0.001	1.936 (1.647–2.276)	< 0.001		1.507 (1.253–1.911)	< 0.001
DM								
MACEs	256 [27.74%]	176 [42.62%]	< 0.001	1.666 (1.375–2.019)	< 0.001		1.408 (1.131–1.754)	0.002
All-cause death	84 [9.10%]	71 [17.19%]	< 0.001	1.968 (1.435–2.700)	< 0.001		1.584 (1.088–2.307)	0.016
Non-DM								
MACEs	611 [22.71%]	504 [36.23%]	< 0.001	1.719 (1.528–1.934)	< 0.001		1.407 (1.233–1.606)	< 0.001
All-cause death	222 [8.25%]	212 [15.24%]	< 0.001	1.932 (1.601–2.333)	< 0.001		1.486 (1.201–1.838)	< 0.001

*CI* confidence interval, *DM* diabetes mellitus, *HR* hazard ration, *Non-DM*, non-diabetes mellitus, *SHR* stress hyperglycemia ratio

*Adjusted for age, SBP, HR, Killip classification, diabetes, hypertension, angina, weight, anterior STE or LBBB, time to treatment > 4 h (TIMI risk score)

**Fig. 2:**
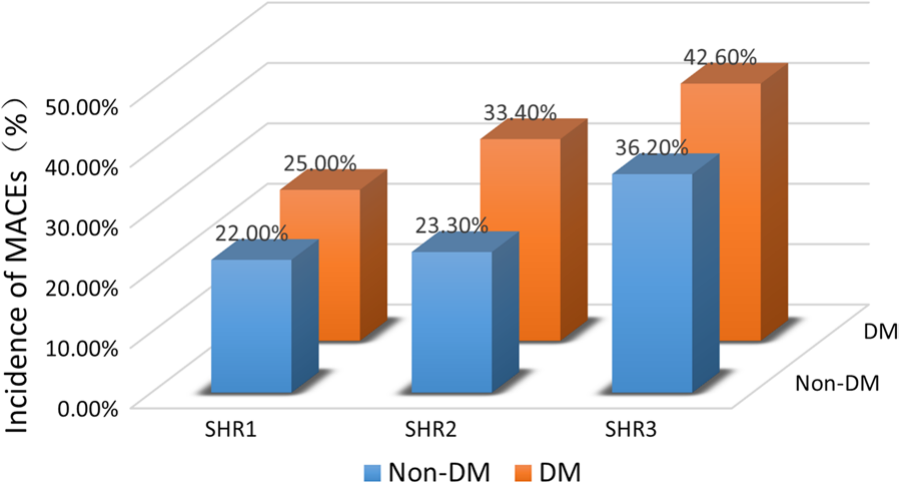
Incidence of MACEs in different subgroups. *DM* diabetes mellitus, *non-DM* non-diabetes mellitus, *MACEs* major cardiovascular adverse events, *SHR* stress hyperglycemia ratio

**Fig. 3:**
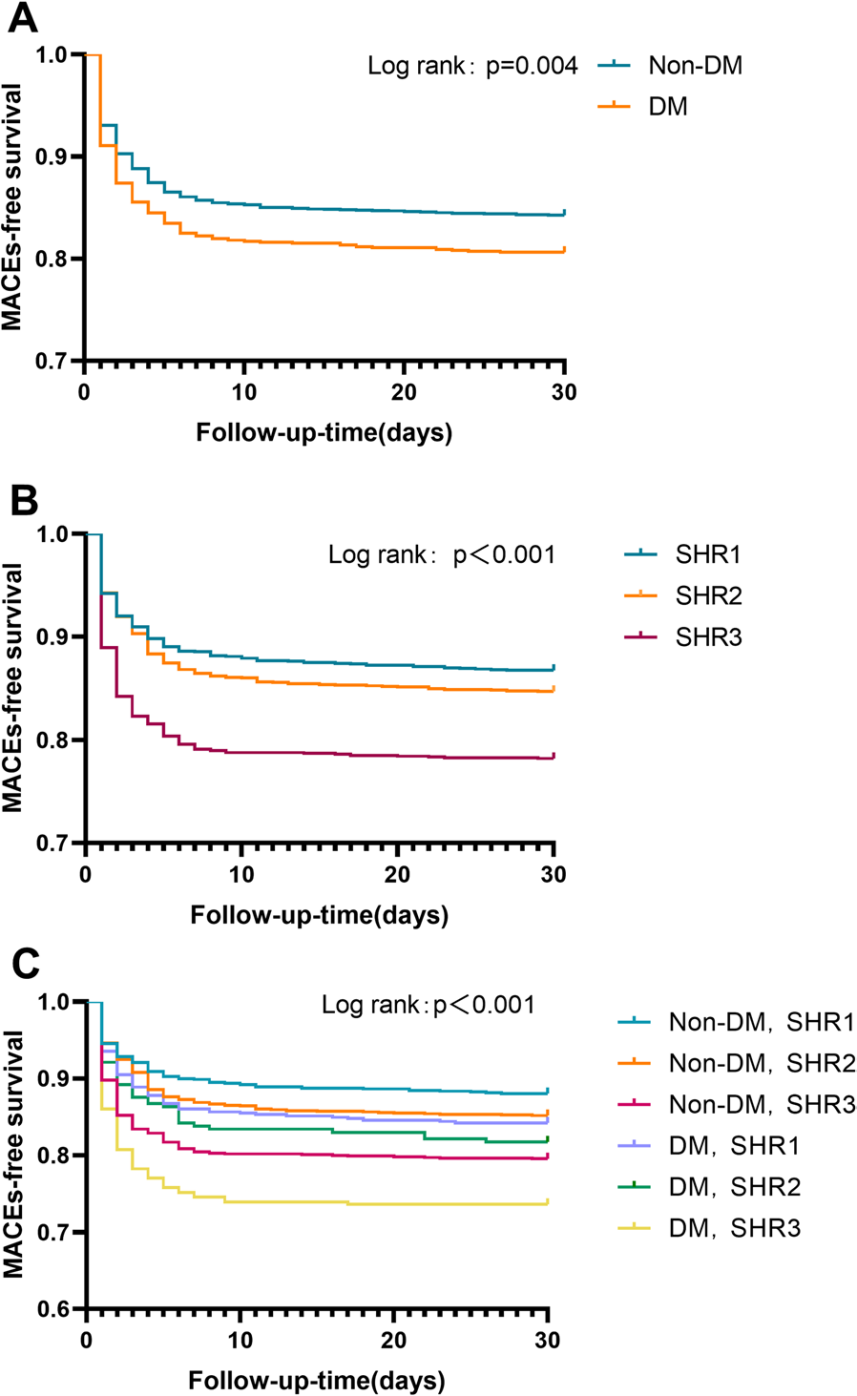
Kaplan–Meier analysis according to diabetes status (**A**), SHR tertiles (**B**), and SHR tertiles with or without diabetes (**C**). *DM* diabetes mellitus, *non-DM* non-diabetes mellitus, *MACEs* major cardiovascular adverse events, *SHR* stress hyperglycemia ratio

The study population was classified into six subgroups for further analysis: SHR1 with and without DM, SHR2 with and without DM, and SHR3 with and without DM groups. Fig. 2 shows the incidence of MACEs in the different groups. The incidence of the MACEs was highest (42.6%) in the SHR3 + DM group. Interestingly, the incidence of MACEs in the SHR3 + non-DM group was even higher than that in the SHR2 + DM group, (36.2% vs. 33.4%). The Kaplan–Meier curves shown in Fig. 3 (A) indicate that patients with acute STEMI and DM had a higher risk of MACEs than their counterparts in the non-DM group (p = 0.004). As Fig. 3 (B) shows, the SHR3 group exhibited the highest risk of MACEs (p < 0.001). However, when considering both SHR tertiles and diabetes status, those with SHR3 and DM had the highest risk of MACEs (p < 0.001). Interestingly, the curve for the SHR3 + non-DM group was positioned just next to that for the SHR3 + DM group.

Univariate Cox regression models suggested that the SHR3 group exhibited a 1.694-fold increase in the risk of MACEs (HR: 1.694, 95% CI 1.532–1.873) and a 1.936-fold increase in the risk of all-cause deaths (HR: 1.936, 95% CI 1.647–2.276) when compared with the SHR1-2 group (all p < 0.001) (Table 2). The p-values, HR and 95% CIs of candidate risk factors derived from TIMI risk score are presented in Additional file 1: Table S1. Predictors with p values < 0.10 were used for adjustment in the multivariable model.

When adjusted for risk factors including age, SBP, HR, Killip classification, DM, hypertension, angina, weight, anterior ST-segment elevation or LBBB, and time to treatment > 4 h, SHR3 was still associated with an increased risk of MACEs and all-cause mortality (all p < 0.05). Within the SHR3 group, both the DM and non-DM subgroups also exhibited an increased risk of MACEs (HR: 1.408, 95% CI 1.131–1.754 and HR: 1.407, 95% CI 1.233–1.606, respectively) and all-cause deaths (HR: 1.584, 95% CI 1.088–2.307 and HR: 1.486, 95% CI 1.201–1.838, respectively) when compared with their counterparts in the SHR1-2 groups (all p < 0.001).

### Predictive value of SHR in the DM and non-DM groups

**Fig. 4:**
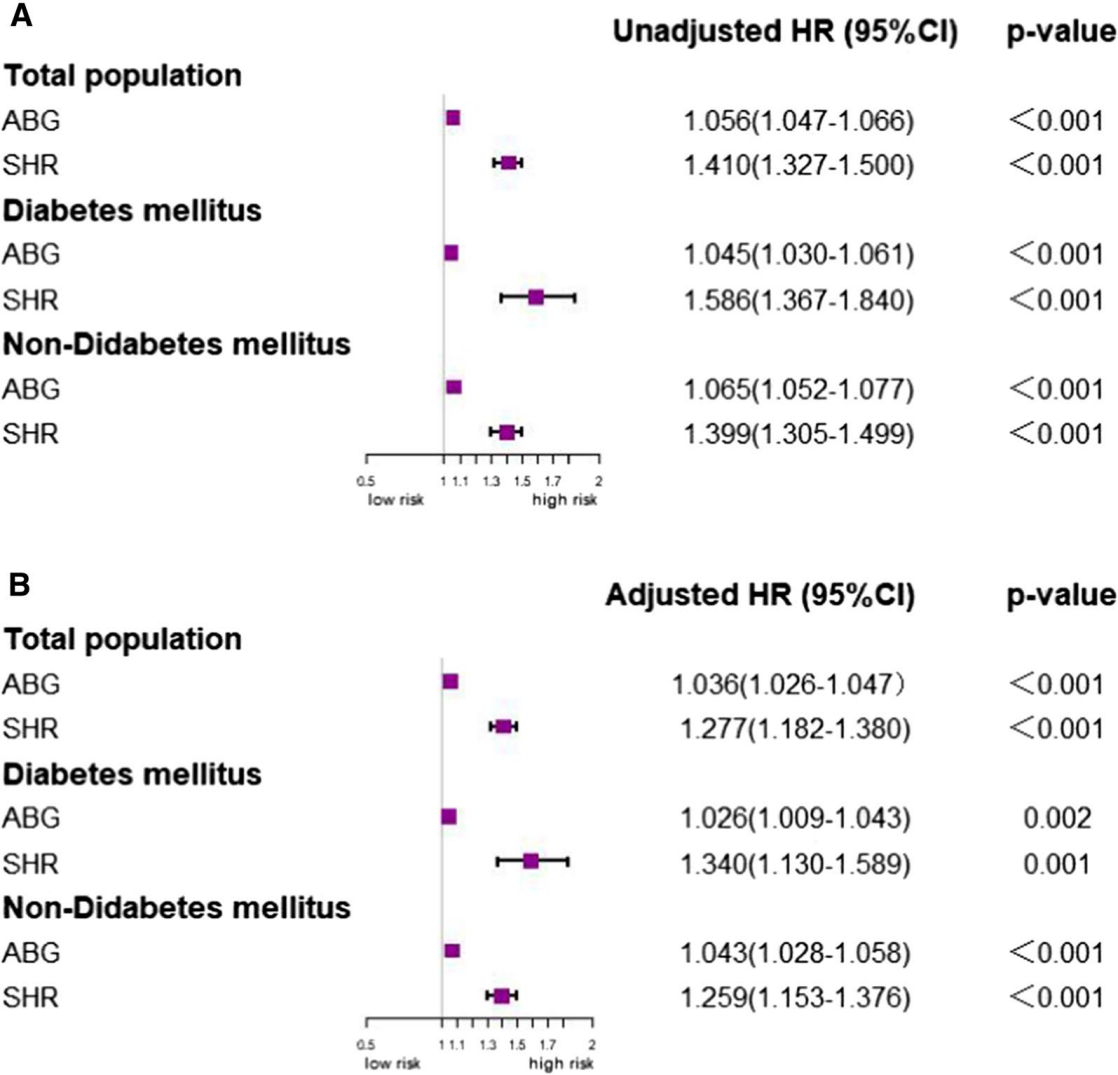
Comparison of ABG and SHR in association with MACEs. **A** Unadjusted model, **B** Adjusted model (Adjusted for age, SBP, HR, Killip classification, DM, hypertension, angina, weight, anterior ST-segment elevation or LBBB, time to treatment > 4 h). *ABG* admission blood glucose, *HR* hazard ratio, *SHR* stress hyperglycemia ratio

Figure 4A shows the unadjusted Cox regression models. In the analysis of the total study population, each 1-SD change in ABG was associated with a 5.6% increased in the risk of MACEs, while each 1-SD increase in SHR was with a 41.0% increased risk for MACEs in the total study population (all p < 0.001). Fig. 4B shows the results of the adjusted Cox regression models, which suggested that each 1-SD change in ABG and SHR was associated with a 2.6% or 34.0% increase in the risk of MACEs in patients with DM, respectively (p = 0.002 and p = 0.001, respectively). For patients without DM, each 1-SD change in ABG and SHR was related to a 4.3% or 25.9% increase in the risk of MACEs, respectively (all p < 0.001).

In multivariable Cox regression models adjusted for age, SBP, HR, Killip classification, DM, hypertension, angina, weight, anterior ST-segment elevation or LBBB, time to treatment > 4 h, the C-statistic values for MACE were 0.765 (0.750–0.781), 0.755 (0.724–0.785), and 0.770 (0.752–0.788) for the total population, DM group, and non-DM group, respectively (Table 3). Moreover, the C-statistics significantly improved when SHR3 was added to the original model; the ΔC-statistics (95% CI) were 0.008 (0.000–0.013) for the total population, and 0.010 (0.003–0.017) for the DM group, and 0.007 (0.002–0.013) for the non-DM group, respectively (all p < 0.05). Table 4 presents the C-statistic values for the adjusted Cox regression models for all-cause mortality. The C-statistics for all-cause mortality improved significantly, reaching 0.006 (0.001–0.010), 0.007 (0.001–0.013), and 0.005 (0.001–0.009) in the total population, DM group, and non-DM group after adding SHR3 into the original models (all p < 0.05).

**Table 3:** C-statistics of SHR3 for predicting MACEs in STEMI

Models	C-statistics (95% CI)	ΔC-statistics (95% CI)	p-value
Total population			
Original model*	0.765 (0.750–0.781)	–	
Original model + SHR3	0.773 (0.758–0.788)	0.008 (0.000–0.013)	0.015
DM			
Original model*	0.755 (0.724–0.785)	–	
Original model + SHR3	0.764 (0.734–0.794)	0.010 (0.003–0.017)	0.008
Non-DM			
Original model*	0.770 (0.752–0.788)	–	
Original model + SHR3	0.777 (0.760–0.794)	0.007 (0.002–0.013)	0.007

* CI* confidence interval, *DM* diabetes mellitus, *Non-DM* non-diabetes mellitus, *SHR* stress hyperglycemia ratio

^*^Original model included age, SBP, HR, Killip classification, diabetes, hypertension, angina, weight, anterior STE or LBBB, time to treatment > 4 h (TIMI risk score)

**Table 4:** C-statistics of SHR3 for predicting all-cause deaths in STEMI

Models	C-statistics (95% CI)	ΔC-statistics (95% CI)	p-value
Total population			
Original modell*	0.773 (0.752–0.795)	–	
Original model + SHR3	0.780 (0.758–0.801)	0.006(0.001–0.010)	< 0.001
DM			
Original modell*	0.795 (0.755–0.835)	–	
Original model + SHR3	0.802 (0.764–0.841)	0.007(-0.001–0.013)	0.031
Non-DM			
Original modell*	0.768 (0.743–0.794)	–	
Original model + SHR3	0.774 (0.748–0.799)	0.005(0.001–0.009)	0.012

*CI* confidence interval, *DM* diabetes mellitus, *Non-DM*, non-diabetes mellitus, *SHR* stress hyperglycemia ratio

*Original model included age, SBP, HR, Killip classification, diabetes, hypertension, angina, weight, anterior STE or LBBB, time to treatment > 4 h (TIMI risk score)

In subgroup analysis, there was no interactions between DM subgroup (odds ratio [OR] for SHR: 1.620; 95% CI: 1.254–2.093) and non-DM subgroup (OR for SHR, 1.538; 95% CI, 1.334–1.773) with the impact of SHR on MACEs (p for interaction = 0.354) (Additional file 1 Table S2).

### Predictive efficiency of the TIMI risk score when considering SHR3 as 1 point

In the ROC analysis, the AUC of the original TIMI risk score for all-cause death in the total study population was 0.760 (Table 5). When SHR3 was used to replace history of DM, hypertension or angina in the original TIMI risk score at a value of 1 point, the Delong test suggested that the AUC significantly improved in the total population and in the DM subgroup (∆AUC 0.009 and 0.010, respectively, all p < 0.05). However, in the non-DM subgroup, ∆AUC improved numerically but not significantly (∆AUC 0.005, p = 0.055). The Hosmer–Lemeshow goodness-of-fit test indicate good fit for each model (all p > 0.05).

**Table 5:** Predictive value of SHR3 added into TIMI risk score for all-cause deaths

TIMI score	Hosmer–Lemeshow (p-Value)	AUC (95%CI)	∆AUC	p-Value
Total population				
Original TIMI score	0.967	0.760 (0.739–0.781)	–	–
SHR3 considered as 1 point	0.948	0.769 (0.748–0.789)	0.009	0.005
DM				
Original TIMI score	0.768	0.784 (0.747–0.822)	–	–
SHR3 considered as 1 point	0.962	0.794 (0.757–0.831)	0.010	0.021
Non-DM				
Original TIMI score	0.978	0.755 (0.730–0.779)	–	–
SHR3 considered as 1 point	0.988	0.759 (0.735–0.784)	0.005	0.055

*AUC* area under curve by receiver-operating characteristic curve analysis*, ∆AUC* difference value of AUC, *CI* confidence interval, *DM* diabetes mellitus, *SHR* stress hyperglycemia ratio, *Non-DM* non-diabetes mellitus

## Discussion

In this study, we evaluated the predictive value of the SHR in patients with acute STEMI. Our findings indicated that the SHR is significantly associated with the risk of MACEs and all-cause mortality in patients with STEMI, among those both with and without DM. Further, the SHR was more effective than ABG in predicting 30-days MACEs. During the 30-days of follow-up period, when patients were stratified into six subgroups according to SHR tertiles and diabetes status, the incidence of MACEs was highest in the SHR3 + DM subgroup. Interestingly, the incidence of MACEs was higher in the SHR3 + non-DM subgroup than in the SHR2 + DM subgroup. Kaplan–Meier curves also showed that the SHR3 + non-DM group exhibited an increased risk of MACEs when compared with the SHR2 + DM subgroup. Notably, a higher SHR index was more strongly associated with the worse prognosis in STEMI than diabetes status or chronic hyperglycemia. Moreover, adding SHR3 into the original models adjusted for factors derived from the TIMI risk score significantly improved the C-statistics. Ultimately, the present study suggests that higher SHR is associated with an increased risk of MACEs in patients with STEMI.

### Stress hyperglycemia and AMI

Stress hyperglycemia involves increases in blood glucose due to sympathetic system activation during critical illnesses such as trauma, sepsis, MI, stroke [11–13]. Calvisi et al. further suggested that, even among patients with COVID-19 pneumonia, DM/stress hyperglycemia is associated higher thromboembolic risk and worse clinical outcomes [11]. Stress-induced increases in the release of glucagon, cortisol and catecholamines promote the rate of glycogenolysis and gluconeogenesis, eventually leading to hyperglycemia [6, 14]. Although the mechanism by which stress hyperglycemia leads to adverse outcomes following critical illness remains unclear, insulin resistance has long been considered a critical defect of stress hyperglycemia [15, 16]. Recently, Garcia Whitlock et al. further reported that forkhead box protein O (FOXO) transcription factors which are involved in the regulation of gluconeogenesis in the liver, represent a predominant driver of stress hyperglycemia via cross-talk between hepatic and adipose-related pathways [17].

Several studies have reported that stress hyperglycemia is independently related to increased mortality and larger infarct size in patients with MI [3, 18, 19]. Paolisso et al. reported that admission hyperglycemia is also an effective predictor of short and long-term prognosis in patients with AMI, including those with MINOCA, indicating that hyperglycemia may play a direct role in microvascular dysfunction [5]. While these data suggest that ABG can be considered a predictor of AMI prognosis, the predictive effectiveness of ABG depends on its definition and the threshold used to characterize stress hyperglycemia. The HORIZONS-AMI trial revealed that the incidence of hyperglycemia upon admission was more predictive of mortality in the non-DM group than in the DM group [18]. This finding supports that hyperglycemia upon admission defined by ABG should be further classified into DM-related hyperglycemia and stress-induced hyperglycemia without diagnosed DM [20].

### Predictive value of SHR in patients with STEMI

To better characterize the relative stress hyperglycemia, Robert et al. proposed the SHR, which combined both the acute and chronic blood glucose status and has been found strongly associated with the risk of adverse outcomes in cases of critical illness [7]. A few recent studies have focused on the ability of the SHR to predict AMI prognosis [21, 22]. However, direct studies concerning the impact of the SHR on the prognosis of STEMI remain limited. Yang et al. investigated the effect of the SHR on MACEs after 30 days of follow up in patients treated via PCI, reporting that SHR was a critical risk factor for MACEs in cases of AMI [21]. However only 13.5% of patients in their study were diagnosed with STEMI. Similarly, Gao et al. conducted a retrospective study of 1,416 patients with STEMI who had undergone PCI [23]. The study endpoints were in-hospital mortality and morbidity without follow-up. In accordance with the current findings, the authors reported that the SHR is a powerful predictor of MACEs in patients with acute STEMI. Notable strengths of our study in relation to previous investigations were the inclusion of 5,417 patients with STEMI in the final analysis and 30-day follow-up for MACEs in all patients.

Marenz et al. used a different formula to calculate chronic glucose levels [8] for 1,553 patients with AMI, including 52% patients with STEMI treated at one center. The authors reported that the ratio of acute-to-chronic glycemia and the ABG exhibited a similar ability to predict in-hospital mortality and morbidity in patients with AMI who had not been diagnosed with DM [8]. Conversely, our study suggested that the predicted efficiency of SHR was better than that of the ABG level in patients with STEMI, regardless of diabetes status (Fig. 4). This difference may be related to the different formulas used and differences in the study populations. Our findings suggest that the SHR is a better predictor of STEMI prognosis than the ABG level. Consistent with the current study, Sia et al. investigated the optimal cut-off value of SHR and ABG for predicting all-cause mortality in patients with AMI who had undergone PCI and found that SHR was better than ABG, regardless of the presence or absence of DM [9]. Şimşek B et al. reported that higher SHR values were associated with an increased risk of no-flow in patients with STEMI after primary PCI and suggested that there were no interactions between SHR and diabetes status [24].

### SHR and TIMI risk score

The TIMI risk score is a traditional tool that is widely utilized to predict short-term STEMI outcomes in clinical situations (e.g., emergency room) given the simplicity of the calculations involved [25]. In the TIMI risk score system, DM history is assigned a value of 1 point. However, in accordance with previous findings [7], our results showed that the risk of MACEs was even higher in the SHR3 + non-DM subgroup than in the SHR2 + DM subgroup, indicating that acute hyperglycemia without DM was also associated with an increased risk of worse prognosis following STEMI. Studies regarding the ability of the SHR to improve the value of the TIMI risk score for predicting all-cause mortality are warranted. We compared the original TIMI risk score with a new score that incorporates SHR3. The AUC analysis for the total population suggested that the predictive value of the tool significantly improved when SHR3 was assigned a value of 1 point to replace the history of DM, angina or hypertension, especially in patients with DM. Subgroup analysis revealed that, in patients with STEMI and non-DM, SHR3 numerically improved the predictive efficiency of the TIMI score, although this change was not significant. Generally, our study provides a new perspective on strategies for improving the clinical value of the TIMI risk score.

### Strengths and limitations

The strengths of the present study were its multi-center observational design and large sample, which included 5,417 patients with acute STEMI from 274 emergency centers. In addition, ABG was determined based on blood glucose levels within 6 ± 2 h after admission, rather than on the results of a random glucose test conducted within the first 24 h, which is more reflecting the true blood glucose level of patients on admission before they received any therapy. We also evaluated whether the SHR can improve the predictive value of the TIMI risk score. However, there were several limitations in this study. First, this was an observational study, indicating that confounders and selection bias may have influenced the study results. Second, the 274 emergency centers included in this study represented different medical levels from rural areas to urban cities throughout China. Given limitations in healthcare at the local level and insufficient implementation of guidelines during the study period, only 12.76% and 50.71% of patients received primary PCI and thrombolytic therapy in this study, respectively. However, in the subgroup analysis (Additional file 1: Table S2), there was no significant difference in the impact of the SHR on MACEs between the PCI (OR for SHR: 1.834; 95% CI 1.029–3.270) and thrombolytic therapy (OR for SHR: 1.432; 95% CI 1.221–1.658) groups (P for interaction = 0.749). Finally, as we focused on the short-term prognosis of acute STEMI over a period of only 30 days, long-term studies are required to verify our results.

## Conclusion

The present results suggest that SHR is independently associated with the risks of MACEs and mortality in patients with STEMI. Furthermore, incorporating the SHR may improve the predictive efficiency of the TIMI risk score in patients with STEMI, especially those with DM.

## Supplementary Information

**Additional file 1**:** Table S1.** Candidate predictors for adjusting multivariable Cox regression analysis for MACEs**.** **Table S2.** Subgroup analysis for associations between SHR and MACEs.

## Data Availability

The datasets generated and analyzed during the current study are not publicly available due privacy and ethical restrictions but are available from the corresponding author on reasonable request.

## Abbreviations

ABG: Admission blood glucose
ACEI: Angiotensin-Converting Enzyme Inhibitors
AMI: Acute myocardial infarction
ARB: Angiotensin Receptor Blocker
AUC: Area under the curve
CI: Confidence interval
DBP: Diastolic blood pressure
DM: Diabetes mellitus
FOXO: Forkhead box protein
GOD: Glucose oxidase
HPLC: High-pressure liquid chromatography
HR: Heart rate
HRs: Hazard ratios
LAD: Left anterior descending
LBBB: Left bundle-branch block
LCX: Left circumflex
LMWH: Low molecular weight heparin
MACEs: Major cardiovascular adverse events
MI: Myocardial infarction
MINOCA: Non-obstructive coronary arteries
OR: Odd ratio
PCI: Percutaneous coronary intervention
RCA: Right coronary artery
ROC: Receiver operating characteristic
SBP: Systolic blood pressure
SD: Standard deviation
SHR: Stress hyperglycemia ratio
STE: ST-segment elevation
STEMI: ST-segment elevation myocardial infarction
TIMI: Thrombolysis in myocardial infarction
