# Supplementary material for: Predictive value of the stress hyperglycemia ratio in patients with acute ST-segment elevation myocardial infarction: insights from a multi-center observational study
Source: Cardiovasc Diabetol. 2022 Mar 29;21:48. doi: 10.1186/s12933-022-01479-8 (PMC8962934; doi:10.1186/s12933-022-01479-8)
Supplement: Supplementary file 1 — Additional file 1: Table S1. Candidate predictors for adjusting multivariable Cox regression analysis for MACEs. Table S2. Subgroup analysis for associations between SHR and MACEs. [file 12933_2022_1479_MOESM1_ESM.docx]

**Additional file material**

**Predictive value of the stress hyperglycemia ratio in patients with acute ST-segment elevation myocardial infarction: Insights from a multi-center observational study**

**Additional file 1: Table S1-S2**

**Additional file 1: Table S1 Candidate predictors for adjusting multivariable Cox regression analysis for MACEs**

| **Variavles** | **HR** | **95% CI** | **p-value** |
| --- | --- | --- | --- |
| Age | 1.043 | 1.038-1.048 | ＜0.001 |
| Weight | 0.984 | 0.980-0.988 | ＜0.001 |
| SBP | 0.990 | 0.988-0.992 | ＜0.001 |
| HR | 1.015 | 1.013-1.018 | ＜0.001 |
| Killip classification | 2.437 | 2.322-2.557 | ＜0.001 |
| Diabetes mellitus | 1.210 | 1.083-1.353 | 0.001 |
| Hypertension | 1.096 | 0.991-1.212 | 0.075 |
| Angina | 1.252 | 1.059-1.481 | 0.009 |
| Anterior STE or LBBB | 1.107 | 1.001-1.223 | 0.047 |
| Time to treatment＞4 hours | 1.164 | 1.044-1.299 | 0.006 |

Abbreviations: HR, heart rate; SBP, systolic blood pressure; STE, ST-segment elevation; LBBB, left bundle-branch block.

**Additional file 1: Table S2 Subgroup analysis for associations between SHR and MACEs**

|  | **Univariable analysis** | | | **Multivariable analysis*** | | | **P-for interaction** |
| --- | --- | --- | --- | --- | --- | --- | --- |
|  | **OR** | **95% CI** | **P-value** | **OR** | **95% CI** | **P-value** |  |
| **DM status** |  |  |  |  |  |  | 0.334 |
| DM | 1.881 | 1.533-2.307 | ＜0.001 | 1.620 | 1.254-2.093 | ＜0.001 |  |
| Non-DM | 1.675 | 1.490-1.883 | ＜0.001 | 1.538 | 1.334-1.773 | ＜0.001 |  |
| **Reperfusion therapy** |  |  |  |  |  |  | 0.749 |
| PCI | 1.968 | 1.323-2.928 | 0.001 | 1.834 | 1.029-3.270 | 0.040 |  |
| Thrombolytic therapy | 1.563 | 1.372-1.781 | ＜0.001 | 1.432 | 1.221-1.658 | ＜0.001 |  |

* Adjusted for age, SBP, HR, Killip classification, diabetes, hypertension, angina, weight, anterior STE or LBBB, time to treatment＞4 hours.

Abbreviations: CI, confidence interval; OR, odd ratio; PCI, percutaneous coronary intervention.
